# A Repurposed Drug for Brain Cancer: Enhanced Atovaquone Amorphous Solid Dispersion by Combining a Spontaneously Emulsifying Component with a Polymer Carrier

**DOI:** 10.3390/pharmaceutics10020060

**Published:** 2018-05-19

**Authors:** Hiroyuki Takabe, Zachary N. Warnken, Yajie Zhang, Daniel A. Davis, Hugh D. C. Smyth, John G. Kuhn, Steve Weitman, Robert O. Williams III

**Affiliations:** 1 Division of Molecular Pharmaceutics and Drug Delivery, College of Pharmacy, University of Texas at Austin, Austin, TX 78712, USA; yjzhang@utexas.edu (Y.Z.); davis.daniel@utexas.edu (D.A.D.); hugh.smyth@austin.utexas.edu (H.D.C.S.); bill.williams@austin.utexas.edu (R.O.W.I.); 2 Division of Pharmacotherapy, College of Pharmacy, University of Texas at Austin, Austin, TX 78712, USA; KUHN@uthscsa.edu; 3 Institute for Drug Development, Cancer Therapy and Research Center (CTRC), University of Texas Health San Antonio, 7979 Wurzbach Dr., San Antonio, TX 78229, USA; Weitman@uthscsa.edu

**Keywords:** atovaquone, hot-melt extrusion, amorphous solid dispersion, supersaturation, glioblastoma multiforme, repurposing drugs

## Abstract

Glioblastoma multiforme (GBM) is the most common and lethal central nervous system tumor. Recently, atovaquone has shown inhibition of signal transducer and activator transcription 3, a promising target for GBM therapy. However, it is currently unable to achieve therapeutic drug concentrations in the brain with the currently reported and marketed formulations. The present study sought to explore the efficacy of atovaquone against GBM as well as develop a formulation of atovaquone that would improve oral bioavailability, resulting in higher amounts of drug delivered to the brain. Atovaquone was formulated as an amorphous solid dispersion using an optimized formulation containing a polymer and a spontaneously emulsifying component (SEC) with greatly improved wetting, disintegration, dispersibility, and dissolution properties. Atovaquone demonstrated cytotoxicity against GBM cell lines as well as provided a confirmed target for atovaquone brain concentrations in in vitro cell viability studies. This new formulation approach was then assessed in a proof-of-concept in vivo exposure study. Based on these results, the enhanced amorphous solid dispersion is promising for providing therapeutically effective brain levels of atovaquone for the treatment of GBM.

## 1. Introduction

Glioblastoma multiforme (GBM) is an aggressive malignant tumor which is both the most common and most lethal central nervous system tumor, accounting for approximately 50% of all primary brain tumors [1]. Despite aggressive treatment with surgical resection, irradiation, and chemotherapy with temozolomide, the median survival rate is around 15 months. The two-year survival with standard care treatment is a mere 26.5 percent [2]. As the name implies, GBM comes in many forms, which adds to the complexity of treatment and resistance to therapies; furthermore, the tumors are topographically diffuse within the brain, making complete surgical resection difficult [3]. Despite the high mortality rate, poor prognosis, and the greater attention in the search for improved therapies, there have been few improvements in treatment. Advancing knowledge of the biology of cancer cells has led to new drug targets for inhibiting cancer. Of these new targets, the signal transducer and activator of transcription 3 (STAT3) has shown to play an important role in the tumorigenesis of many cancer types, including GBM [4,5]. STAT3 is activated in GBM and cancer stem cells of other brain tumors [4,6]. The activation of STAT3 is associated with a worse prognosis and more aggressive disease than those with lower levels of activated STAT3 [7,8,9]. Inhibition of STAT3 has been reported to inhibit proliferation of GBM cells and attenuate resistance to temozolomide, which has translated into survival benefits based on in vivo models [10,11,12,13,14,15]. 

An improved understanding of the molecular and cellular targets for cancer treatment, including STAT3, in combination with high-throughput screening, has enabled several opportunities for repurposing drugs for the treatment of cancers [16]. Atovaquone has recently received attention as a candidate for repurposing from its current indications for the prevention and treatment of malaria and *Pneumocystis jiroveci* pneumonia (PCP) for the treatment of solid tumors [17,18,19]. Specifically, atovaquone has been shown to be a potent STAT3 inhibitor, an inhibitor of OXOPHOS, and to increase radiosensitivity of tumors [20,21,22]. To the best of our knowledge, the activity of atovaquone against GBM has not been reported in the literature.

Atovaquone (MW-367 Da) is a poorly water soluble, highly lipophilic compound (less than 0.2 μg/mL, log P 5.31) [23]. Currently, there are two commercially approved formulations of atovaquone: Mepron^®^, a micronized suspension that reportedly has the higher absolute bioavailability of the two, of about 47% when administered with food [24,25]. The bioavailability of these products is greatly affected by the presence of food. For example, the bioavailability of atovaquone from Mepron^®^ administered without food is reduced by about 50%. In addition to this significant limitation, the plasma concentration of atovaquone does not increase proportionally with increases in dose, likely as a result of its very poor dissolution in the gastrointestinal tract. For the treatment of GBM, therapeutically effective brain concentrations of atovaquone will be required to inhibit tumor growth. However, even after four-times daily dosing for two weeks, the cerebrospinal fluid concentration in HIV-infected children was found to be less than 1% of the plasma concentrations, which resulted in levels below 1 µM. While generally regarded as a safe drug, there have been reports of an increase in hepatic enzymes after administration with atovaquone [26]. Previous formulation strategies to improve the bioavailability and brain concentrations of atovaquone, including a nanosuspension formulation and nanoemulsion formulation, have been reported in the literature [27,28,29]. While the nanosuspension product appears to produce brain levels sufficient for the treatment of toxoplasmic encephalitis, the levels are below those likely needed for brain cancer treatment. The nanoemulsion formulation required greater than 20% of the formulation to contain dimethyl sulfoxide in order to dissolve a sufficient quantity of atovaquone, which exceeds the values that have currently been used previously in any FDA-approved product [29]. Therefore, there is a need for a formulation strategy that uses excipients known to be safe in humans in order to improve the bioavailability of atovaquone to achieve therapeutically effective brain concentrations for the treatment of glioblastoma. 

Amorphous solid dispersions present a promising strategy for improving the bioavailability of poorly soluble drugs [30,31,32]. Solid dispersions can be prepared by thermal methods (e.g., melting and hot-melt extrusion (HME)) [33,34,35], solvent evaporation [36,37], and supercritical fluid technology [38,39,40]. Binary solid dispersions of highly lipophilic drugs such as atovaquone and polymeric carriers are limited in their drug loading capacity and therefore their feasibility due to dissolution rate limitations [41]. The present study introduces a new formulation approach that overcomes the limitations of binary solid dispersions made by thermal processing containing lipophilic drugs by the inclusion of a spontaneously emulsifying component (SEC). The SEC component contains surfactant, cosurfactant, and an oil phase such that when thermally processed with the polymer and drug, improved wetting and rapid dispersion of the formulation, either from granules or tablets, into fine particles (e.g., <2.5-micron diameter) of atovaquone allowed for rapid drug dissolution and absorption. This resulted in enabling therapeutically effective brain levels of atovaquone to be achieved.

The hypothesis of the present study is that formulating atovaquone as an enhanced amorphous solid dispersion will lead to increased brain concentrations of atovaquone, permitting it to be repurposed for the treatment of GBM. The activity of atovaquone against GBM is determined from cell viability studies with four GBM cell lines. These results are then used to determine therapeutically effective brain concentrations for atovaquone. We propose that the incorporation of an SEC into the atovaquone/polymeric carrier composition by thermal processing will result in rapid disintegration and dispersion of the atovaquone formulation into fine particles that are available for rapid dissolution and absorption. These improved properties from the formulation will translate to achieving therapeutically effective brain levels to treat GBM.

## 2. Materials and Methods 

### 2.1. Materials

Atovaquone was purchased from Molekula (Irvine, CA, USA). Benzyl benzoate and polyethylene glycol (PEG) (average mol weight 200) were purchased from Sigma Chemical Co. (St. Louis, MO, USA). Isopropyl palmitate was purchased from Spectrum Laboratory Products, Inc. (New Brunswick, NJ, USA). Mineral oil, propylene glycol, and oleic acid were purchased from Fisher Scientific (Pittsburgh, PA, USA). Affinisol HPMC HME 15 LV was kindly donated from Dow Pharma & Food Solutions (Midland, MI, USA). Super-refined polysorbate 80-LQ-(MH), super-refined polysorbate 20-LQ-(MH), and super-refined PEG 400-LQ-(MH) were kindly donated from CRODA Inc. (East Yorkshire, UK). Kollidon VA64, Kollidon 30, and Cremophor RH 40 were kindly donated from BASF Corp. (Florham Park, NJ, USA). Capmul MCM NF and Captex 300 NF were kindly donated from Abitec Corp. (Jamesville, Rock County, WI, USA). Capryol 90, Transcutol HP, Labrafac lipophile WL 1349, Labrafil M 2125 CS, and Labrasol were obtained from Gattefosse SAS (Paramus, NJ, USA). All other chemical reagents used were of analytical grade. 

### 2.2. Atovaquone Solubility in Pharmaceutical Excipients Used in the Formulations 

Excess atovaquone (100 mg/mL) was added to each of the following excipients: benzyl benzoate, Tween 20, Tween 80, PEG 200, PEG 400, Labrafac, Labrafil, Capryol, Capmul, Captex, propylene glycol, and isopropyl palmitate. The mixtures were shaken at 100 rpm at 37 °C for 48 h. Each individual sample was centrifuged at 3000 rpm for 10 min. The supernatant was then filtered through a 0.45 µm PTFE filter. Quantification of atovaquone content in the filtrate was performed using high-performance liquid chromatography (HPLC). The HPLC method was conducted as follows: it was performed using a Dionex Ultimate 3000 (Thermo Fisher Scientific Inc., Waltham, MA, USA) with an Inertsil^®^ ODS-2 column (5 µm, 4.6 × 250 mm) with a mobile phase 0.1% trifluoroacetic acid (TFA) in acetonitrile: 0.1% TFA in deionized water (80:20) at flow rate of 2 mL/min and a run time of 10 min and detection by UV absorbance at 251 nm.

### 2.3. Confirming the Composition of the Spontaneously Emulsifying Components

To determine the ratios useful as an SEC, compositions were made by mixing the solvent, surfactant, and lipid at varying ratios to create a ternary phase diagram. 10 µL of each formulation was added to 10 mL of water and gently agitated by inverting the contents several times. Emulsification was determined by a Zetasizer Nano ZS (Malvern Instruments, Worcestershire, UK), and the criteria used to define an acceptable SEC were: monodisperse distribution, z-average droplet size <500 nm, and no physical separation of phases by visual observation. 

### 2.4. Atovaquone Solubility in Each of the Spontaneously Emulsifying Components

Excess atovaquone (100 mg/mL) was added to each SEC: SEC 1 (Tween 80: Capmul MCM NF: Captex 300 NF = 0.5:0.25:0.25), SEC 2 (Cremophor RH 40: Capmul MCM NF: Captex 300 NF = 0.5:0.25:0.25), SEC 3 (Labrasol: Capmul MCM NF: Captex 300 NF = 0.9:0.05:0.05), and SEC 4 (Benzyl benzoate: Tween 20: PEG 400 = 0.3:0.4:0.3). The mixtures were shaken at 100 rpm at 37 °C for 48 h. Each individual sample was centrifuged at 3000 rpm for 10 min. The supernatant was then filtered through a 0.45 µm PTFE filter. Quantification of atovaquone content in the filtrate was determined by HPLC. 

### 2.5. Flory–Huggins Theory and Miscibility of Compositions

The Flory–Huggins theory was used as a preformulation tool to develop phase diagrams to predict the miscibility and physical stability of atovaquone and pharmaceutically acceptable polymer combinations [42,43]. The free energy of mixing ($\Delta G_{mix})$ is related to the temperature (*T*), volume fraction of the drug (Φ), the relative size of the polymer compared to the drug (*m*), as well as the interaction parameter (χ). The χ value stems from the nonideal entropy of mixing of the pharmaceutically acceptable polymer molecule with the therapeutic agent and takes into account the contribution from the enthalpy of mixing [44]. According to the Flory–Huggins theory,

(1) $$\Delta G_{mix}\;=RT\left(\Phi_{drug}ln\Phi_{drug}+\frac{\Phi_{polymer}}{m}ln\Phi_{polymer}+\chi \Phi_{drug}\Phi_{polymer}\right)$$

where Φ is the volume fraction, χ is the *F*−*H* interaction parameter, *R* is the molar gas constant, and *T* is the temperature. *m* is the ratio of the volume of a pharmaceutically acceptable polymer to the therapeutic agent molecular volume and is estimated based on Equation (2).

(2) $$m\;=\;\frac{\frac{MW_{polymer}}{\rho_{polymer}}}{\frac{MW_{drug}}{\rho_{drug}}}$$

where *MW_polymer_* and *MW_drug_* are the molecular weights of the pharmaceutically acceptable polymer and therapeutic agent, respectively, and ρ*_polymer_* and ρ*_drug_* are the densities of the pharmaceutically acceptable polymer and therapeutic agent, respectively. The χ value was estimated by analyzing the melting point depression and melting enthalpy of the drug (∆*H_fus_*) of blends of atovaquone and polymer, obtained using differential scanning calorimetry (DSC). Following analysis of melting point depressions, the χ value can be calculated using the following rearranged equation [45]:

(3) $$\left(\frac{1}{T_{M}^{mix}}-\frac{1}{T_{M}^{pure}}\right)\left(\frac{\Delta H_{fus}}{-R}\right)-ln\Phi_{drug}-\left(1-\frac{1}{m}\right)\Phi_{polymer}=\chi \Phi_{polymer}^{2}$$

where *T_M_* values are the melting points of the mixture of the pure therapeutic agent, *R* is the ideal gas constant, ∆*H_fus_* is the heat of fusion for the pure therapeutic agent, *m* is a constant for the relative size of the pharmaceutically acceptable polymer to the therapeutic agent, and Φ represents the volume fraction of the therapeutic agent or pharmaceutically acceptable polymer. The slope of the best-fit line of the plotted Equation (3) is considered to be equivalent to χ. By understanding χ as a function of temperature, metastable and unstable regions for the combination can be predicted by generating a spinodal (boundary between unstable and metastable regions) and binodal (boundary between metastable and stable regions) curves [46]. 

Based on this, atovaquone (ranging from 45 to 95%, *w*/*w*) was mixed with PVP K30, Kollidon VA 64, or Affinisol HPMC HME 15 LV including 4% Tween 20, 3% PEG 400, and 3% benzyl benzoate; and the melting point depression was determined by differential scanning calorimetry (DSC; DSC Q20, TA Instruments, New Castle, DE; 5 mg samples, ramped at 10 ℃/min from 30 to 250 ℃). Melt depressions obtained were analyzed using the Flory–Huggins theory (following) to calculate a χ-value (interaction parameter). The Flory–Huggins theory was applied to generate the Gibbs free energy of mixing. In this study, a mixture of polymer and SEC was handled as one polymer and analyzed. 

### 2.6. Preparation of Atovaquone Formulations by Hot-Melt Extrusion 

For formulations containing an SEC, each of the individual components were weighted and vortexed prior to being added to the atovaquone and PVP K30. This composition of the SECs used in the formulations is presented in Table 1. The formulations were processed using a HAAKE Minilab II microextruder with a screw speed of 150 rpm at 180 °C. The cooled extrudate was milled into granules (<600 µm). A control formulation (ATQ + POL) was made that contained only atovaquone and polymer.

### 2.7. Characterization of Amorphous Solid Dispersions

#### 2.7.1. X-Ray Diffractometry (XRD) 

The powder samples (bulk atovaquone; formulations 1, 2, 3, 4; and control ASD) were investigated using an X-ray diffraction instrument: Miniflex 600 (Rigaku, Woodlands, TX, USA). The instrument was operated at an accelerating voltage of 40 kV and 15 mA. All samples were subjected to the same program: scanned range of 0–50° at a step size of 0.05°/s and scan speed of 2°/min.

#### 2.7.2. Differential Scanning Calorimetry (DSC)

The thermal properties of the formulations were assessed using modulated DSC (DSC Q20, TA Instruments, New Castle, DE, USA). Approximately 5 mg of each sample was loaded into a standard aluminum pan. Experiments were performed at a heating ramp rate of 3 °C/min in the range of 40–250 °C, with a modulation temperature amplitude of 1 °C/min and nitrogen purge of 50 mL/min.

#### 2.7.3. In Vitro Dispersion Test for Tablets

**Figure 1:**
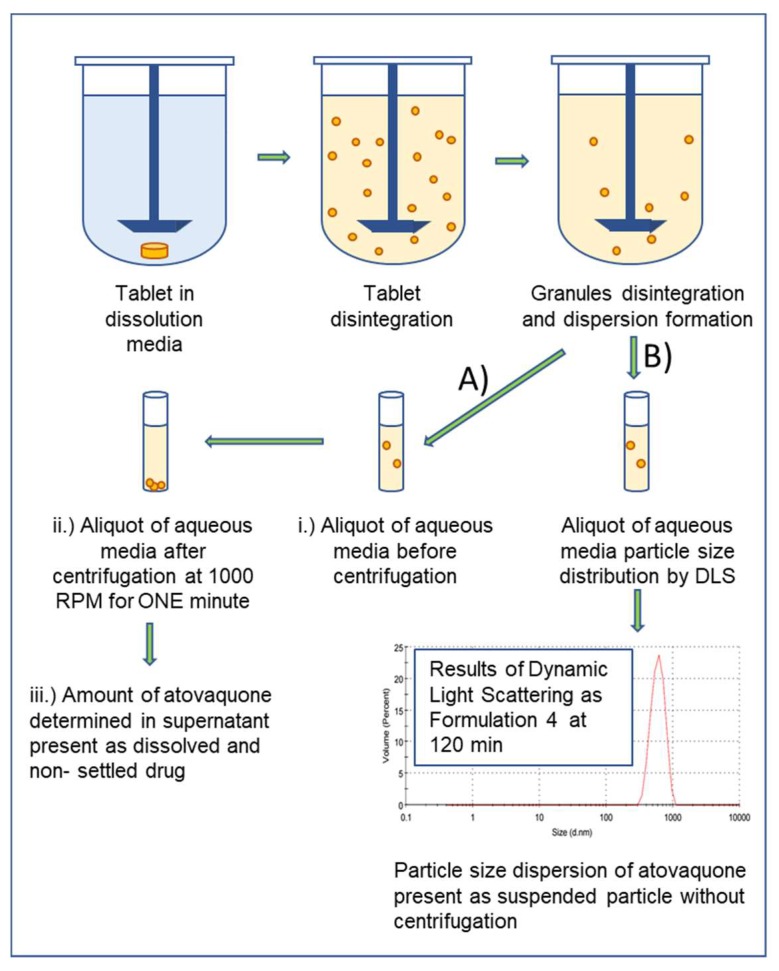
Schematic diagram of the atovaquone tablet dispersibility test to indicate the efficiency of the atovaquone tablet or granules to disintegrate and disperse into nonaggregated fine particles (low micron size range) in the aqueous media. (A) represents the amount of atovaquone determined by HPLC, that is: (i) dissolved in the aqueous media; (ii) dissolved in the emulsion droplets formed when the SEC from the formulation is mixed with the aqueous media; and (iii) remains suspended as fine particles in the low micron size range (<2–3 microns) in the aqueous media after removal of an aliquot from the vessel and centrifuging it for one minute at 1000 rpm. (B) represents the particle size distribution resulting from disintegration of the atovaquone tablet and subsequent granule disintegration, as measured by dynamic light scattering (DLS) of the aqueous media sampled at each timepoint, without centrifugation.

The ability of tablets to disperse into nonaggregated fine particles (in the low micron range) in an aqueous environment, which were made by compressing granules (300 mg) of each formulation into tablets (1000 BAR, 10.0 mm thickness), was determined in deionized water using an SR8 Plus dissolution tester (Hanson Research Corp., Chatsworth, CA, USA) equipped with minipaddles. Figure 1 describes the methodology for assessing the dispersibility of the tablets. Specifically, analyzing the dispersibility of the tablets was performed as follows: Dispersibility test setup: paddle speed 75 rpm; 900 mL water; 37 °C; media aliquots were sampled at each time point, centrifuged for 1 min at 1000 rpm, diluted with ACN, and assayed using HPLC. Test samples were studied in triplicate, with 1 mL samples withdrawn from the vessels at 5, 10, 15, 30, 60, 120, 240 and 360 min. The particle size was determined for each aliquot at the following time points: initial, 30, 60, 120, 240, 360 min, using a Zetasizer Nano ZS (Malvern Instruments, Worcestershire, UK). Dispersibility is reported as the particle size of the aqueous media as a function of time.

#### 2.7.4. In Vitro Dispersion Test for Granules

Dispersibility of granules was performed as described above and as follows: Dispersibility test setup: paddle; 900 mL water; 75 rpm; 37 °C; media aliquots were sampled at each time point, centrifuged for 1 min at 1000 rpm, diluted with acetonitrile, and assayed by HPLC as described above (Figure 1). Test samples were studied in triplicate, with 1-mL samples withdrawn from the vessels at 5, 10, 15, 30, 60, 120, 240, and 360 min. The particle size was determined for each noncentrifuged aliquot at the following time points: initial, 30, 60, 120, 240, and 360 min, using a Zetasizer Nano ZS (Malvern Instruments, Worcestershire, UK). The upper limit of measurement using the Zetasizer Nano ZS is about 10 microns. Dispersibility is reported as the particle size measured in the dissolution media without centrifugation as a function of time.

#### 2.7.5. Biphasic Dissolution (Octanol/Water) Test

The biphasic dissolution method was used to test the dissolution performance of the atovaquone formulations in order to provide results that may better reflect the relative in vivo bioavailability of the formulations [47,48,49,50]. Drug dissolution of atovaquone dissolved from granules was performed as follows: Dissolution test setup: paddle; 200 rpm; 37 °C, 200 mL octanol/700 mL water; media aliquots were sampled from the octanol layer, all samples were immediately filtered through 0.22 µm PTFE syringe filters, diluted with can, and assayed by HPLC as described above. Test samples were studied in triplicate, with 1 mL samples withdrawn from the vessels at 5, 10, 15, 30, 60, 120, 240, and 360 min.

#### 2.7.6. Cell Culture Conditions

GBM cell lines U87-MG, LN-18, and SF-188 were cultured in 1:1 DMEM/F12 (GIBCO, Carlsbad, CA, USA) supplemented with 10% fetal bovine serum (Corning, Manassas, VA, USA) and 1% penicillin and streptomycin. GBM cell line SJ-GBM2 was cultured in Iscove’s Modified Dulbecco’s Media (GIBCO, Carlsbad, CA, USA) supplemented with 20% fetal bovine serum, Insulin-Transferrin-Selenium supplement (Corning, Bedford, MA, USA), and 1% penicillin and streptomycin (ATCC, Manassas, VA, USA). Cell cultures were maintained at 37 °C in humidified air containing 5% CO_2_. 

#### 2.7.7. Cell Viability Test in Glioblastoma Multiform Cell Lines

Atovaquone was dissolved in DMSO and diluted with the respective complete media to the target concentration for each cell line. Cells were seeded at 5000 cells per well for each cell line. U87-MG and SF-188 cell lines were treated with atovaquone 24 h after seeding. LN-18 and SJ-GBM2 were treated with atovaquone 36 h after seeding. After treatment plates were incubated at 37 °C in humidified air containing 5% CO_2_ for 48 h, followed by analysis by MTT assay (Invitrogen, Carlsbad, CA, USA). 

#### 2.7.8. Proof-of-Concept Drug Exposure Study

Animal studies were performed in accordance with the University of Texas at Austin Institutional Animal Care and Use Committee. Female C57Cl/6 mice were dosed by oral gavage at 100 mg/kg atovaquone dose with either formulation 1 or control ASD. Mice were sacrificed 1 h after dosing and serum and brains were collected from the mice, immediately frozen, and stored at −80 °C until analysis. 

In order to test the effect of the SEC on the performance of the formulations in vivo, formulation 1 or control ASD formulations were loaded at 3 mg per capsule into size M capsules (Torpac Inc., Fairfield, NJ, USA), resulting in an atovaquone dose of approximately 20 mg/kg in C57Cl/6 mice. Mice were sacrificed at 0.25, 1, 2, 4, 8, and 12 h, and their serum was collected and immediately frozen at −80 °C until analysis. 

#### 2.7.9. Analysis of Atovaquone Concentrations in the Brain and Serum

Brain tissue was weighed and diluted 10 µL/mg tissue with a 75% methanol solution. Tissue was homogenized at 4500 rpm using a rotor stator homogenizer (Kinematica Polytron, Luzern, Switzerland), followed by centrifugation at 13,000× *g* for 6 min and analysis of the supernatant. Serum (100 µL) was precipitated with 1 mL of 0.1% formic acid acetonitrile, followed by shaking for 30 min and centrifugation at 13,000× *g* for 6 min. Supernatant from brain samples were analyzed for atovaquone concentration using a high-performance liquid chromatograph (Shimadzu LC-20AD pump, SIL-20AC HT Autosampler, CTO-20AC Column Oven; Shimadzu Scientific Instruments, Columbia, MD, USA) equipped with a tandem mass spectrometry detector (ABSciex 400Q Trap Mass Spectrometer; AB Sciex, Framingham, MA, USA) monitored in negative multiple reaction monitoring (MRM) mode for precursor/product ions: *m/z* 364.9/336.9 Da. Mobile phases A (0.1% formic acid in Millipore water) and B (0.1% formic acid in acetonitrile) were eluted at 0.5 mL/min flow rate using a C8 column (Mac-Mod ACE 3 C9 50 × 3 mm; Mac-Mod Analytical, Chadds Ford, PA, USA) and a gradient method: 35% mobile phase B from 0 to 1 min, followed by ramping mobile phase B from 35 to 98% from 1 to 5 min. Mobile phase B was held at 98% from 5 to 7 min, followed by ramping down to 35% from 7 to 10 min. The retention time for atovaquone was 5.87 min with this gradient. Supernatant from serum samples were analyzed for atovaquone concentration by high-performance liquid chromatography (Dionex Ultimate 3000; Thermo Fisher Scientific Inc., Waltham, MA, USA) with a Microsorb-MV^®^ 100 ^®^ (5 µm, 4.6 × 150 mm) with a mobile phase of 0.1% TFA in acetonitrile: 0.1% TFA in deionized water (80:20) at flow rate of 1 mL/min, a run time of 10 min, and detection by UV absorbance at 251 nm.

## 3. Results and Discussion

### 3.1. Atovaquone Is Effective against Glioblastoma Multiforme Cell Lines

**Figure 2:**
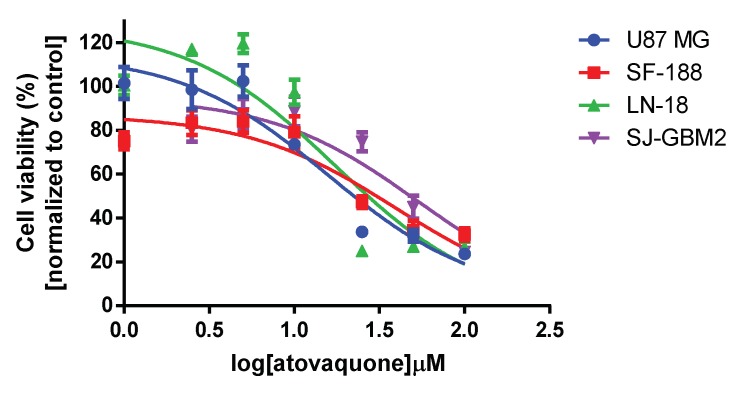
Atovaquone decreased cell viability of glioblastoma multiforme (GBM) cell lines (represented by the various colors) in a dose-dependent manner after incubation for 48 h. Error bars represent the standard deviation from *n* = 6 repeats.

The concentrations for inhibition of tumor growth were determined by cell viability studies in four GBM cell lines. Figure 2 depicts the cell viability as a result of increasing atovaquone concentration. The IC50/IC90 values for the U87-MG, LN-18, SF-188, and SJ-GBM2 cell lines were 15.9/142.7, 17.4/156.6, 39.1/351.9, and 53.3/479.7, respectively, after normalizing for the effect of the required DMSO concentration on the cell lines. 

Atovaquone activity in glioblastoma multiforme has not been previously reported. The activity of atovaquone on the cell lines varied from cell line to cell line; however, it was shown to be effective in concentrations similar those of the standard care treatment, temozolomide [51]. In addition to the direct activity that atovaquone had on these cell lines, there is evidence from other STAT3 inhibitors that the effects may be more pronounced in the presence of temozolomide [15,52,53]. Furthermore, a STAT3 inhibitor has been shown to increase the sensitivity of temozolomide-resistant GBM to temozolomide, which may subsequently decrease the recurrence of GBM after resection [14]. While the concentrations needed to inhibit the cell lines were similar to those reported for temozolomide, its viability as a treatment for GBM is directly dependent on the brain concentrations attainable for atovaquone as achievable by the administered formulation.

Unfortunately, commercially approved atovaquone products have low bioavailability due to the poor water solubility and high lipophilicity of atovaquone. These products, as well as those experimental formulations reported in the literature, show ineffectively low brain concentrations of atovaquone, insufficient for GBM treatment [25,27,28,29]. Amorphous solid dispersions are a promising formulation strategy for improving the solubility of poorly water-soluble drugs [54]. However, binary compositions of amorphous solid dispersions containing highly lipophilic drugs are typically limited in their drug-loading capacity due to decreased dissolution performance [41]. To overcome these limitations of binary amorphous solid dispersions for atovaquone, addition of the SEC component to the formulation enhanced the performance of the dosage form, including dispersibility of the dosage form in a form that promotes dissolution and which translates to improved brain levels after administration. 

### 3.2. Developing the Atovaquone Formulations

#### 3.2.1. Four Spontaneously Emulsifying Components Selected for the Formulations

Atovaquone’s solubility in several pharmaceutically acceptable excipients is shown in Table 2. Atovaquone exhibited the highest solubility in benzyl benzoate (14.31 mg/mL). The solubility of atovaquone in the surfactants studied was about 10 mg/mL or less. Atovaquone solubility in each lipid and cosurfactant (solvent) studied was also low. 

Specific excipient combinations were studied in order to select SEC compositions with differing solubilities for atovaquone, resulting in the four SEC compositions reported in this paper. The ratios of each component in the SECs are shown in Table 1. The criteria for the excipient combination to be a suitable SEC for atovaquone were that a monodisperse droplet size distribution of the emulsified SEC droplets by dynamic light scattering was formed and that no physical separation of the phases was observed visually after dilution with water. The results shown in Table 2 confirm that the average droplet size (d_v50_) of the SEC dispersed in water for formulations 1, 2, 3, and 4 were 122.4 nm, 64.1 nm, 186.1 nm, and 51.0 nm, respectively. The droplet size distribution observed for each of these SEC formulations was monodisperse. The solubility of atovaquone in each SEC formulation 1, 2, 3, and 4 at 37 °C was 8.7 ± 0.5 mg/mL, 8.6 ± 0.4 mg/mL, 12.5 ± 0.1 mg/mL, and 13.5 ± 0.6 mg/mL, respectively (Table 2). The solubility of atovaquone in each SEC composition was much greater than its solubility in water (0.2 μg/mL) [55]. 

Different formulations, each exhibiting a different solubility for atovaquone, were selected to assist in discerning the mechanisms by which the SEC enhances the performance of the formulations. Atovaquone was added into the thermally processed formulations at an amount that was several times in excess of atovaquone’s solubility in the SEC. Therefore, the formulations containing the SEC are not solid self-emulsifying drug delivery systems (SEDDS), where the drug is completely dissolved in the emulsifying component [56,57,58,59]. The formulations with SECs exhibiting different levels of atovaquone solubility allowed us to discriminate whether our formulations were SEDDS and an amorphous solid dispersion combined or something new from the overall combination of excipients and processing. 

#### 3.2.2. PVP K30 Selected as the Polymer Carrier in the Thermally Processed Formulation 

**Figure 3:**
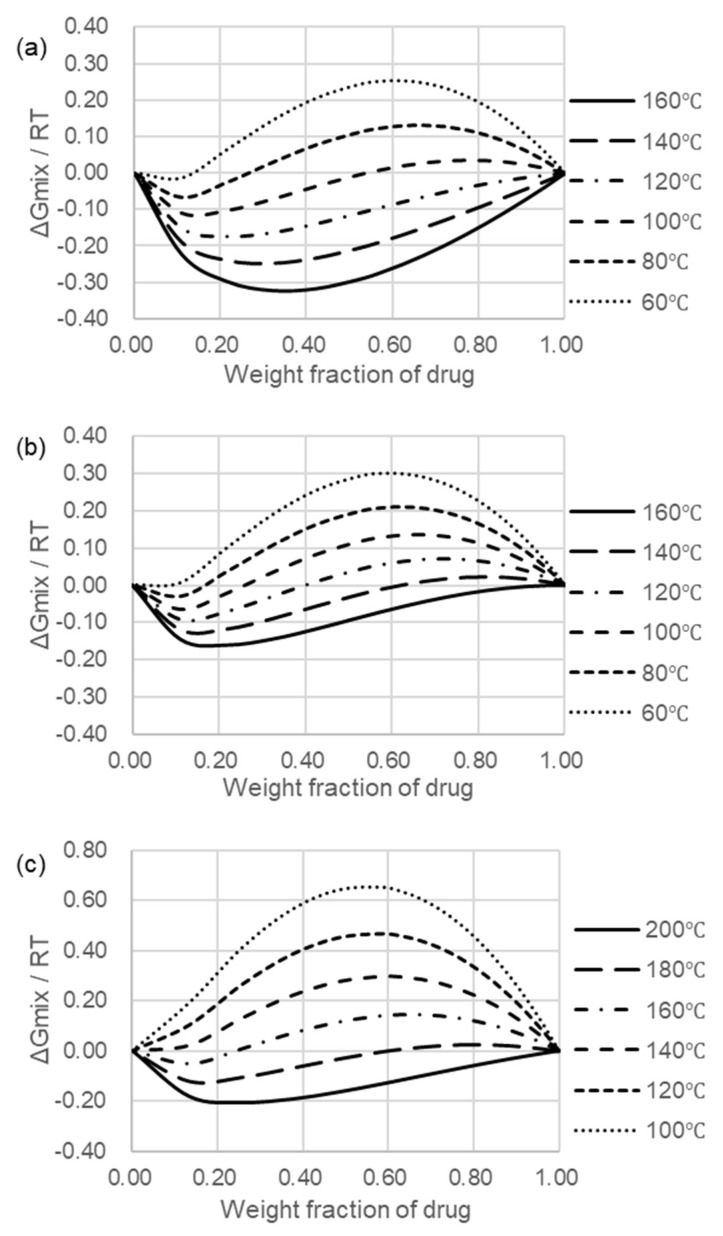
Gibbs free energy of mixing (ΔGmix) diagrams of atovaquone in polymer carriers with the SEC from formulation 4 present. (**a**) Atovaquone and PVP K30. (**b**) Atovaquone and Kollidon VA64. (**c**) Atovaquone and Affinisol 15LV.

In order to develop an amorphous solid dispersion with adequate drug load and physical stability, the drug–polymer miscibility was estimated using Flory–Higgins modeling. The free energy of mixing, ΔGmix, for increasing drug fractions in three polymers at several temperatures relevant for hot-melt extraction is presented in Figure 3. 

PVP K30 was chosen as the polymer carrier as it showed the greatest miscibility with atovaquone of the polymers tested. When the ΔGmix/RT is negative, the miscibility between two components is favored (Figure 3a). PVP K30 showed negative values at relatively low temperature conditions. In addition, PVP K30 showed similar negative values even at higher drug loading, indicating miscibility of atovaquone and PVP K30.

### 3.3. Solid State Characterization of the Thermally Processed Formulations 

#### 3.3.1. Hot-Melt-Extrusion-Processed Formulations Are Amorphous by Powder X-Ray Diffractometry (XRD) 

**Figure 4:**
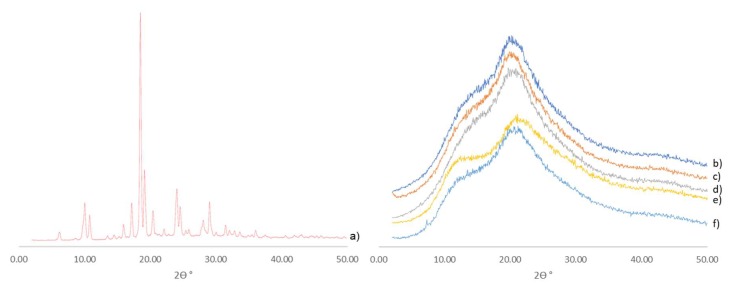
X-ray diffraction results for (a) bulk atovaquone, (b) formulation 1, (c) formulation 2, (d) formulation 3, (e) control ASD, and (f) formulation 4.

The results from XRD are shown in Figure 4. Atovaquone bulk material (Figure 4a) exhibited its characteristic crystalline peaks whereas a broad halo profile was observed for formulations 1, 2, 3, 4, and control ASD (see Figure 4b–f), thus indicating a lack of crystallinity. 

#### 3.3.2. The Glass Transition Temperature of Formulations Containing the SEC is Lower Than That of the Control Formulations 

**Figure 5:**
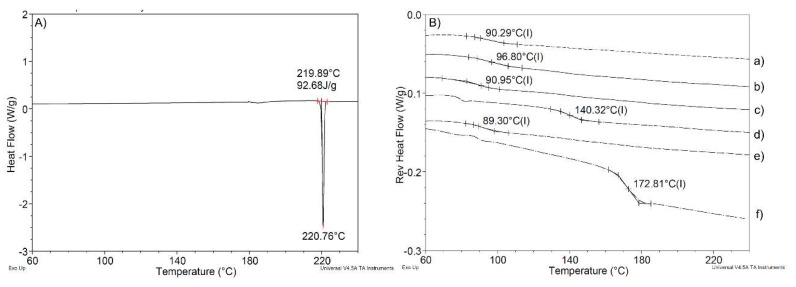
Differential scanning calorimetry results for (**A**) bulk atovaquone from a plot of heat flow (W/g) versus temperature (°C) and (**B**) glass transition temperature (Tg (s)) for compositions of (a) formulation 1, (b) formulation 2, (c) formulation 3, (d) control ASD, (e) formulation 4, and (f) bulk PVP K30 obtained from a plot of reverse heat flow (W/g) versus temperature (°C).

Thermograms obtained by modulated DSC of each of the compositions tested as described in Table 1 are shown in Figure 5. The melting point of atovaquone was found to be about 220 °C (Figure 5A). PVP K30 (Figure 5B) exhibited a glass transition temperature of about 170 °C. The thermogram characterizing control ASD (Figure 5B(d)) shows plasticization from the inclusion of atovaquone. Further plasticization was observed with the inclusion of the SECs in formulations 1, 2, 3, and 4. 

The SEC containing formulations could exhibit less physical stability for certain polymer and drug combinations due to an increase in molecular mobility of the drug in the carrier. The decrease in the glass transition temperature as well as the observation of only a single glass transition temperature in the thermograms is evidence of the compatibility of the SEC components with the polymer carrier system. In the particular case with atovaquone and PVP K30, the glass transition temperatures remained at a temperature that is sufficiently high to predict a physically stable system [60]. 

### 3.4. The Extent and Rate of Dissolution Is Greater When the SEC Is Present in the Formulation

**Figure 6:**
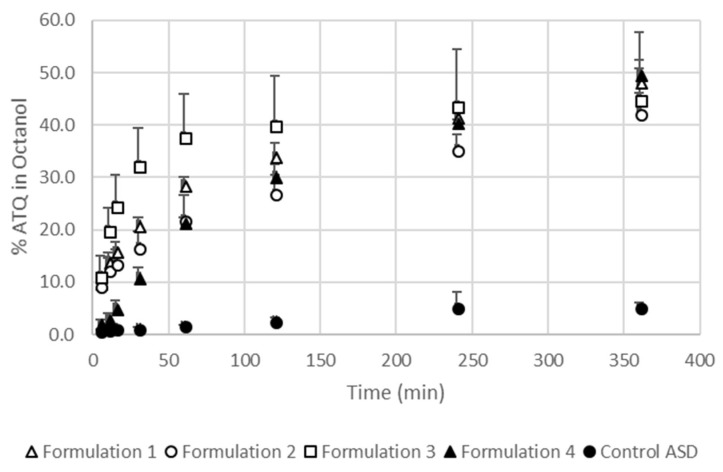
Result of biphasic dissolution (octanol/water) test performed on formulation 1, formulation 2, formulation 3, formulation 4, and control ASD in 200 mL octanol/700 mL water at 37 °C. Results from *n* = 3. Error bars represent the standard deviation. ASD – Atovaquone.

Drug dissolution of atovaquone in biphasic media from granules (Figure 6) was rapid for all formulations containing any of the SEC compositions (formulation 1, 2, 3, and 4). The dissolution rate of formulation 3 was fastest at the beginning of the test, but the dissolution rate decreased and became similar to the other SEC-containing formulations after 240 min. The dissolution rate of formulation 3 peaked at the point where it exceeded 40%. At 360 min, the percentage of atovaquone in the octanol layer of the formulations containing the SEC was between 40% and 50%. The control granules with no SEC (control ASD) showed a low amount of atovaquone dissolved across all times, which resulted in less than 10% of atovaquone measured in the octanol layer.

During the dissolution of drug from amorphous solid dispersions, supersaturation followed by crystallization of the drug in the dissolution media can occur and is influenced by the degree of supersaturation [61]. The biphasic dissolution method provides both a sink condition for atovaquone that reflects the drug being absorbed by passive diffusion in the gastrointestinal tract as well as nonsink conditions for dissolution of the drug in the aqueous phase. The four formulations containing SEC, each with varying degrees of solubility for atovaquone, were shown to perform similarly in the biphasic dissolution test. The dissolution performance was not different for the formulations with the SEC containing a higher solubility for atovaquone, showing that the SEC does not behave like a SEDDS, where the drug is dissolved in the emulsion droplets before and after dilution in an aqueous environment [62]. However, based on the equilibrium solubility of atovaquone in the SECs, the maximum percentage of atovaquone in the formulation that could be dissolved in the SEC is between about 0.4% and 0.7%. As the droplet size of the SEC upon dilution and solubility of atovaquone in the SEC are not shown to affect their performance in the range tested, we hypothesized that the improved performance during the in vitro biphasic dissolution test was related to the enhanced wetting and ability to disperse into fine particles of the formulation containing an SEC, an observation confirmed visually during the biphasic dissolution test.

### 3.5. Formulations Containing the SEC Rapidly Wet and Disperse into Fine Particles from Direct Compressed Tablets and Granules

**Figure 7:**
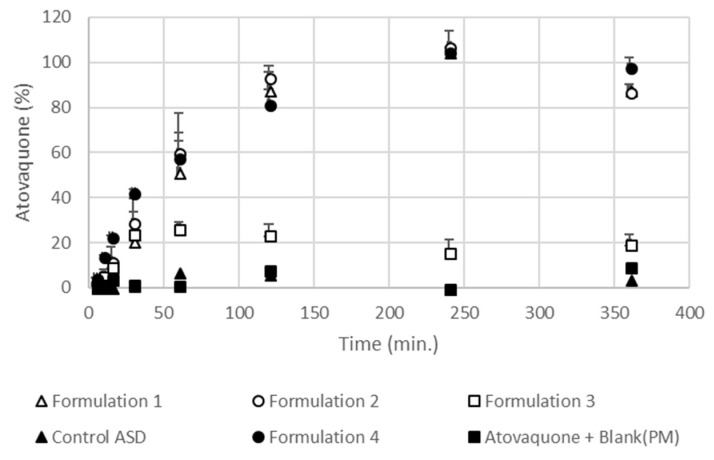
Result of tablet dispersibility tests (described in Figure 1) of formulations 1, 2, 3, and 4; control ASD; and bulk atovaquone mixed with the blank (Table 1) physical mixture (PM) in 900 mL water at 37 °C.

**Figure 8:**
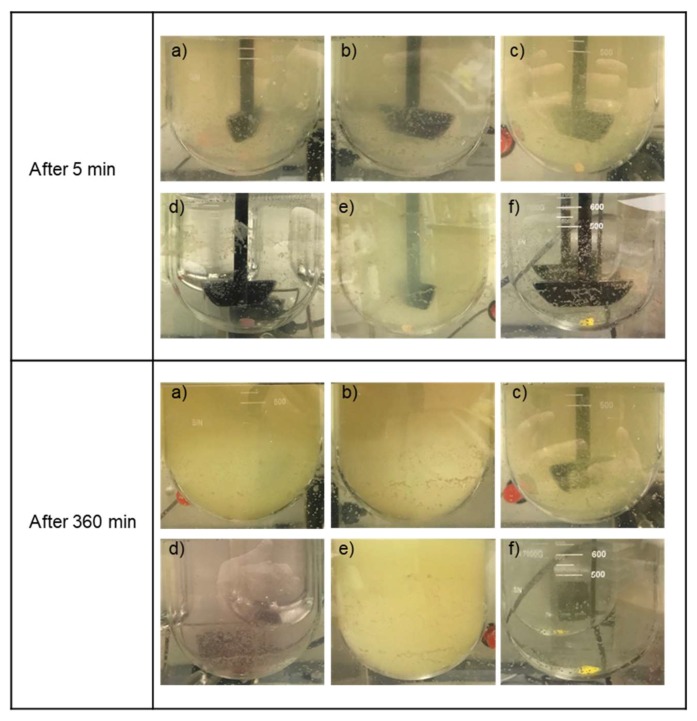
Photographs of tablet dispersibility test as a function of time: (**a**) formulation 1, (**b**) formulation 2, (**c**) formulation 3, (**d**) control ASD, (**e**) formulation 4, and (**f**) atovaquone mixed with the blank (Table 1).

To investigate the ability of the formulations to wet and disperse into fine, nonaggregated particles into the low micron size range, in vitro dispersion tests were performed on each formulation prepared as either directly compressed tablet and granule forms. The results showing the extent of atovaquone from tablets that were either dissolved or dispersed as fine, nonsettling, nonaggregated particles is presented in Figure 7. All except for one of the tableted formulations containing an SEC resulted in similar extents of dispersion, reaching over 80% being present as either particles less than 2.5 µm or being dissolved within 120 min. These values were much higher than those found for the tested control formulations, which were less than 10%. Formulation 3 had visually larger particles in the aqueous media during the dispersion studies from the tablet dosage form that resulted in a lower extent of dispersed drug. The size of the resulting particles during the tablet dispersion studies qualitatively correlated well with the amount of atovaquone in the supernatant as either dissolved or fine, nonsettled, nonaggregated particles (Table 3). Visually, the differences in the wetting and dispersion of atovaquone from the tested formulations are illustrated in Figure 8. 

The resulting size of the particles in the water during each study comparing the various SECs and control formulations is shown in Table 3. Particle size results were only able to be determined for particular formulations and time points due to the size cutoff limitations for dynamic light scattering (DLS) instruments. The size of the particles in the media during the tablet dispersion studies were found to be about 0.7–3 µm for formulations containing SECs, except that of formulation 3, whose particles at later time points were visually observable and therefore too large to analyze by DLS. Particle sizes of either control formulations, solid dispersion without a SEC, or physical mixture with crystalline atovaquone were also found to be visually observable and therefore could not be determined using DLS.

**Figure 9:**
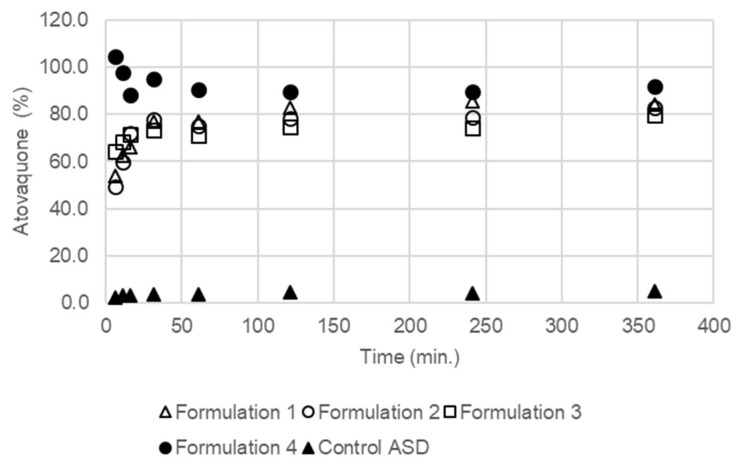
Result of dispersibility tests for granules (described in Figure 1) of formulations 1, 2, 3, and 4; and control ASD in 900 mL water at 37 °C.

Also, dispersibility studies on granules of the formulations described in Table 1 were performed. Figure 9 shows the difference in the amount dispersed between the different formulations. Similar to the dispersion studies performed on tablets, formulations containing the SECs rapidly dispersed into particles that remained suspended upon centrifugation or were dissolved. In the case of the experiments with granules, all formulations with the SECs behaved similarly, with nearly 80% dispersed at 120 min. The control solid dispersion without a SEC wetted and sank to the bottom of the vessel; however, it remained as large granules which failed to distribute throughout the aqueous media. The resulting particle sizes of the dispersed formulations were smaller than those found during the tablet dissolution studies. Formulations containing a SEC had particles sized between 0.4 and 0.9 µm. The size of the resulting particles was dependent on the composition of the SEC, with the smallest particles resulting from dispersing of formulation 3, containing the highest ratio of surfactant in the formulation (Table 3). As with the dispersibility studies performed on the tablets, the particles of the control solid dispersion formulation without a SEC (control ASD) was visually observable and therefore could not be determined by DLS. 

Dispersion tests have been used to study the performance of many self-emulsifying drug delivery systems in the past [63,64], and were utilized in this study to assess the total amount of fine, nonsettling, nonaggregated particles after being subjected to relatively small centrifugal forces to account for drug dispersed as fine particles, in SEC droplets, or dissolved in the media. In both the tablet and granule studies, a significantly greater amount of atovaquone was found to be finely dispersed or dissolved compared to the control formulations, with the exception of the formulation 3 tablets. Future testing on the resulting tablet properties which result from the different SEC formulations may explain the failure of formulation 3 to finely disperse in a similar manner to the other formulations when in a tablet, but not when in granules. These results support our hypothesis, showing that the enhanced dissolution performance is related to the ability of the formulations to disperse. 

### 3.6. Preliminary Studies Confirm that SEC-Containing Formulations Lead to Effective Brain Levels Identified for Glioblastoma Multiforme Treatment

Formulation 1 and control ASD were tested by oral gavage in a proof-of-concept study in mice. A single time point at 1 h post-dosing was investigated at a dose of 100 mg/kg for each formulation to characterize the amount of atovaquone that reached the brain after dosing with each formulation. With six mice in the formulation 1 group and five in the control ASD group, brain levels were found to be 37.2 ± 38.0 µmol/kg and 26.7 ± 25.5 µmol/kg, respectively.

Although atovaquone showed in vitro activity against GBM cell lines, in order to effectively treat GBM, atovaquone must cross the blood–brain barrier and achieve and maintain concentrations in the brain high enough to be therapeutically effective. Previous studies have explored brain levels after oral dosing of atovaquone suspension and a nanosuspension with equivalent doses as used in our study, but failed to achieve feasible levels in the brain tissue. In these studies previously reported, repeated dosing for several days was performed and resulted in brain levels of around 1 µmol/kg [27,28]. Based on our results in four glioblastoma cell lines, this low level is likely insufficient for GBM treatment. Atovaquone brain levels from our proof-of-concept study in mice provides promise for achieving sufficient brain levels to treat GBM once steady-state concentrations are reached [25]. 

Brain concentrations after dosing with the formulation containing no SEC (control ASD) were 28% lower than those found for formulation 1. The SEC component also provides a benefit, as illustrated in the in vitro studies, in the wetting and dispersion of the granule and tablet formulations for likely improved absorption. In the case of the oral gavage studies in this study, the granule formulation was predispersed and was suspended in a liquid medium in order to dose at levels that are comparable to human dosing equivalents. To achieve this dose in mice, predispersing the control ASD formulation in normal saline using high shear forces before dosing was performed. From the in vitro tests, we expect the benefits of the SEC-containing formulations to be more pronounced in the final dosage form, such as a tablet or capsule, where the formulation must be wetted, disintegrated, and dispersed in the gastric milieu.

Future studies are planned to confirm steady-state brain concentrations of atovaquone after dosing with the formulations (not predispersed) as well as studies that test the final dosage form of these formulations to assess differences in their performance in vivo. 

### 3.7. The Formulation Containing an SEC Leads To Increased Exposure after Dosing in Mice 

**Figure 10:**
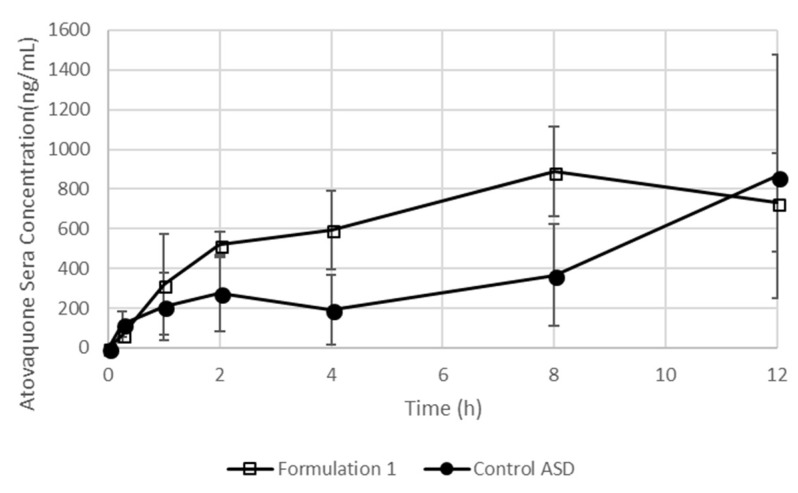
Atovaquone concentration in sera after dosing with formulation 1 and control ASD formulation in size M capsules at a dose of 20 mg/kg. Data are a mean of three mice, with error bars representing the standard deviation.

Formulation 1 and control ASD were loaded into capsules to test the effect of the improved dispersibility formulation (formulation 1) on the in vivo exposure to atovaquone. Formulation 1 resulted in a quicker rise in atovaquone concentrations and a shorter Tmax of 8 h compared to control ASD (Figure 10). The AUC_0–12h_, calculated by the trapezoidal method, after dosing with formulation 1, was significantly higher than that of the control ASD formulation: 7880.7 ± 158.6 ng·h/mL compared to 4422.2 ± 592.6, respectively (*p* value < 0.01).

As the in vitro tests showed the enhanced performance of the SEC-containing formulations was related to the wetting and dispersibility of the formulations, animal dosing in capsules instead of as a predispersed suspension was used to evaluate the differences in the formulation from a pharmacokinetic point of view. Based on the nearly 1.8-fold higher AUC_0–12h_ of the SEC-containing formulation, we conclude that the enhanced performance of formulation 1 seen in vitro translates to the in vivo context. The need for testing both a predispersed preparation for brain levels as well as the capsule preparation for testing in vivo performance is due to the dose-loading limitations caused by the maximum filling amount in the size M capsules; in this case, 3 mg per capsule. 

## 4. Conclusions

In conclusion, atovaquone has the potential to be efficacious against GBM; however, previously reported formulations and those currently marketed in the United States are limited in the amount of atovaquone available in the brain for activity due to poor bioavailability. By formulating atovaquone into a binary amorphous solid dispersion containing polymers by hot-melt extrusion, atovaquone showed low and inadequate dissolution performance during the biphasic dissolution test. The incorporation of a SEC into the amorphous solid dispersion resulted in much quicker dissolution, which was shown to be a result of the rapid dispersion of the formulation into fine particles when introduced into an aqueous environment. The proof-of-concept results in mice provides promise for achieving atovaquone brain levels that will be sufficient for the treatment of GBM. Future preclinical studies to assess the toxicity of atovaquone after the increase in exposure due to the improved formulation are planned. Based on the reported pharmacokinetics of atovaquone in humans, relatively early clinical trials testing the new atovaquone formulation efficacy to extend GBM patients’ lives is a feasible endeavor. 

## Figures and Tables

**Table 1:** The composition of each formulation investigated containing atovaquone. The formulations contain PVP K30 and a spontaneous emulsifying component.

	Thermally Processed Formulations by Hot-Melt Extrusion (% *w*/*w*)					
	Formulation 1	Formulation 2	Formulation 3	Formulation 4	Control ASD (No SEC)	Blank (No Drug)
Atovaquone	20	20	20	20	20	
PVP K30	70	70	70	70	80	90
Spontaneously Emulsifying Component Composition						
Benzyl benzoate	-	-	-	3	-	3
PEG 400	-	-	-	3	-	3
Capmul MCM NF	2.5	2.5	0.5	-	-	-
Captex 300 NF	2.5	2.5	0.5	-	-	-
Tween 20	-	-	-	4	-	4
Tween 80	5	-	-	-	-	-
Cremophor RH 40	-	5	-	-	-	-
Labrasol	-	-	9	-	-	-

ASD: Amorphous Solid Dispersion ; SEC: Spontaneously Emulsifying Component.

**Table 2:** Results of the solubility study for atovaquone in each of the excipients investigated making up the spontaneously emulsifying component (SEC) described in Table 1. The solubility study was conducted at 37 °C. Results are from *n* = 3 replicates.

Excipient and Excipient Combinations	Solubility of Atovaquone ± Standard Deviation (mg/mL)	Droplet Size Distribution (d_v,50_; nm) for SEC Diluted in Water (10 μL of SEC Diluted into 10 mL of Water)
Benzyl benzoate	14.3 ± 0.4	-
PEG 400	7.2 ± 0.7	-
Capmul MCM NF	3.5 ± 0.3	-
Captex 300 NF	4.6 ± 0.6	-
Tween 20	9.7 ± 0.7	-
Tween 80	10.2 ± 1.2	-
Labrasol	10.2 ± 0.5	-
SEC for formulation 1	8.7 ± 0.5	122.4
SEC for formulation 2	8.6 ± 0.4	64.1
SEC for formulation 3	12.5 ± 0.1	186.1
SEC for formulation 4	13.5 ± 0.6	51.0

Solubility of atovaquone in water = 0.2 μg/mL [23]; Solubility of atovaquone in Cremophor RH 40 could not be measured at 37 °C.

**Table 3:** Particle size distribution as Z-average resulting from disintegration of the atovaquone tablet and subsequent granule disintegration.

Dosage Form	*Z* = Average Particle Size (μm)						
	Time (min)		30	60	120	240	360
**Tablet**	**Formulation**	**1**	2.7	2.6	2.1	1.7	1.6
		**2**	2.3	1.6	1.5	1.2	1.2
		**3**	1.4	1.6	- ^2^	- ^2^	- ^2^
		**4**	0.86	0.72	0.70	0.59	0.65
		**Control ASD**	- ^2^	- ^2^	- ^2^	- ^2^	- ^2^
		**5 ^1^**	- ^2^	- ^2^	- ^2^	- ^2^	- ^2^
**Granule**		**1**	0.87	0.79	0.74	0.68	0.73
		**2**	0.81	0.61	0.59	0.56	0.54
		**3**	0.39	0.40	0.39	0.38	0.36
		**4**	0.49	0.49	0.48	0.46	0.47
		**Control ASD**	- ^2^	- ^2^	- ^2^	- ^2^	- ^2^
		**5 ^1^**	- ^2^	- ^2^	- ^2^	- ^2^	- ^2^

^1^ Formulation 5 as a physical mixture. ^2^ Over the particle size range for the instrument (0.3 nm–10 µm), there are visible particles.

## Declaration

This work in part has been previously presented as a poster entitled “Atovaquone Amorphous Solid Dispersions Improved by Combining SEDDs and Hot Melt Extrusion” at the 2017 American Association of Pharmaceutical Scientists Annual Meeting.
